# Supplementary material for: The Tudor SND1 protein is an m6A RNA reader essential for replication of Kaposi’s sarcoma-associated herpesvirus
Source: eLife. 2019 Oct 24;8:e47261. doi: 10.7554/eLife.47261 (PMC6812964; doi:10.7554/eLife.47261)
Supplement: Supplementary file 1. — The number of unique peptides sequences and peptide spectrum matches (PSM’s) assigned to each protein as identified by mass spectrometry is displayed for each bait. [file elife-47261-supp1.pptx]

## Slide 1
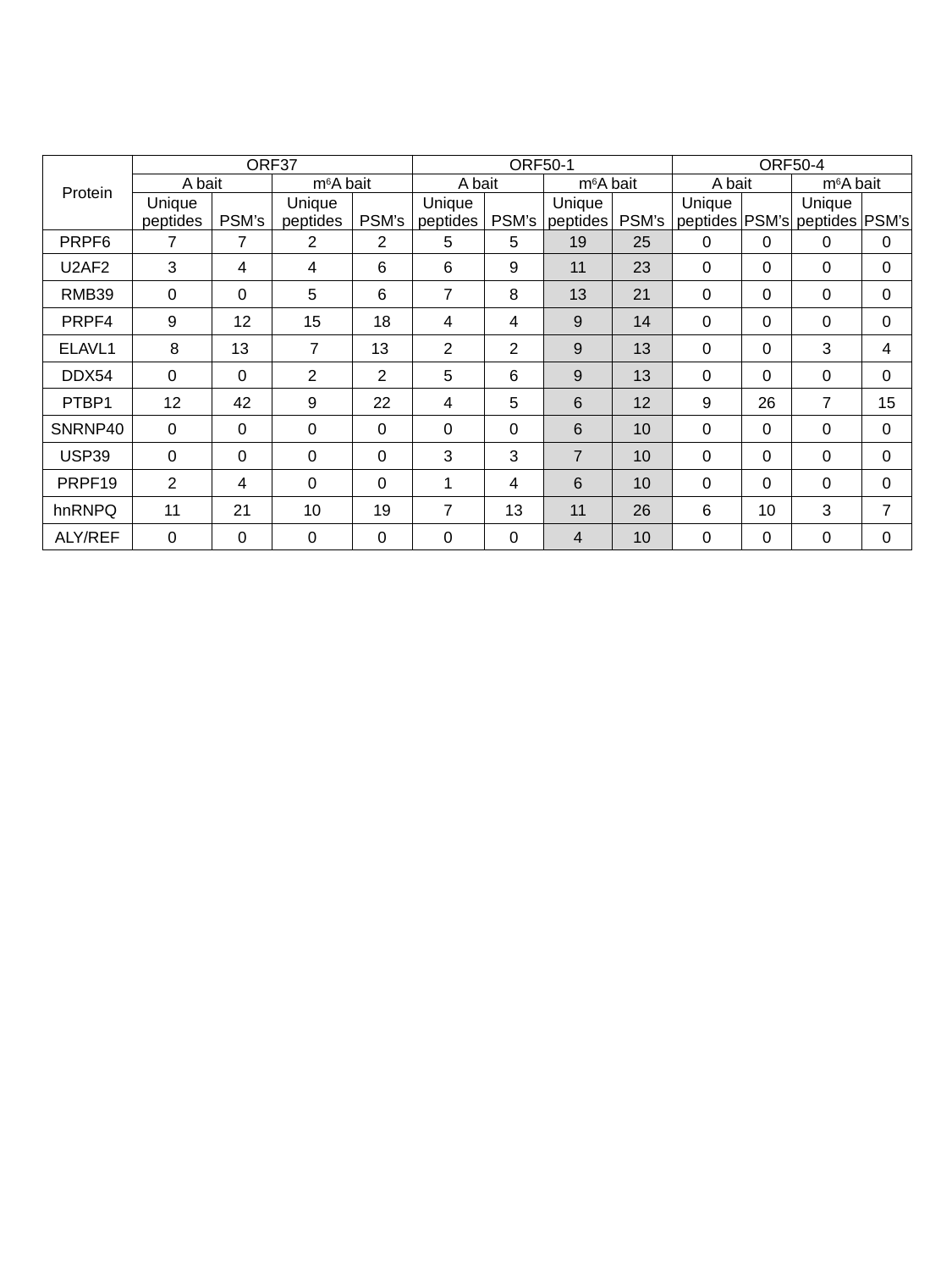

| Protein | ORF37 | | | | ORF50-1 | | | | ORF50-4 | | | |
| --- | --- | --- | --- | --- | --- | --- | --- | --- | --- | --- | --- | --- |
| | A bait | | m6A bait | | A bait | | m6A bait | | A bait | | m6A bait | |
| | Unique peptides | PSM’s | Unique peptides | PSM’s | Unique peptides | PSM’s | Unique peptides | PSM’s | Unique peptides | PSM’s | Unique peptides | PSM’s |
| PRPF6 | 7 | 7 | 2 | 2 | 5 | 5 | 19 | 25 | 0 | 0 | 0 | 0 |
| U2AF2 | 3 | 4 | 4 | 6 | 6 | 9 | 11 | 23 | 0 | 0 | 0 | 0 |
| RMB39 | 0 | 0 | 5 | 6 | 7 | 8 | 13 | 21 | 0 | 0 | 0 | 0 |
| PRPF4 | 9 | 12 | 15 | 18 | 4 | 4 | 9 | 14 | 0 | 0 | 0 | 0 |
| ELAVL1 | 8 | 13 | 7 | 13 | 2 | 2 | 9 | 13 | 0 | 0 | 3 | 4 |
| DDX54 | 0 | 0 | 2 | 2 | 5 | 6 | 9 | 13 | 0 | 0 | 0 | 0 |
| PTBP1 | 12 | 42 | 9 | 22 | 4 | 5 | 6 | 12 | 9 | 26 | 7 | 15 |
| SNRNP40 | 0 | 0 | 0 | 0 | 0 | 0 | 6 | 10 | 0 | 0 | 0 | 0 |
| USP39 | 0 | 0 | 0 | 0 | 3 | 3 | 7 | 10 | 0 | 0 | 0 | 0 |
| PRPF19 | 2 | 4 | 0 | 0 | 1 | 4 | 6 | 10 | 0 | 0 | 0 | 0 |
| hnRNPQ | 11 | 21 | 10 | 19 | 7 | 13 | 11 | 26 | 6 | 10 | 3 | 7 |
| ALY/REF | 0 | 0 | 0 | 0 | 0 | 0 | 4 | 10 | 0 | 0 | 0 | 0 |
