# Supplementary material for: The Tudor SND1 protein is an m6A RNA reader essential for replication of Kaposi’s sarcoma-associated herpesvirus
Source: eLife. 2019 Oct 24;8:e47261. doi: 10.7554/eLife.47261 (PMC6812964; doi:10.7554/eLife.47261)
Supplement: Supplementary file 2. — While proteins with methyl-transferase activity (highlighted in bold) were recruited to methylated viral baits, neither m6A indirect readers nor IGF2BP proteins were enriched in any of the viral baits. The number of unique peptides sequences assigned to each protein as identified by mass spectrometry is displayed for each bait. [file elife-47261-supp2.pptx]

## Slide 1
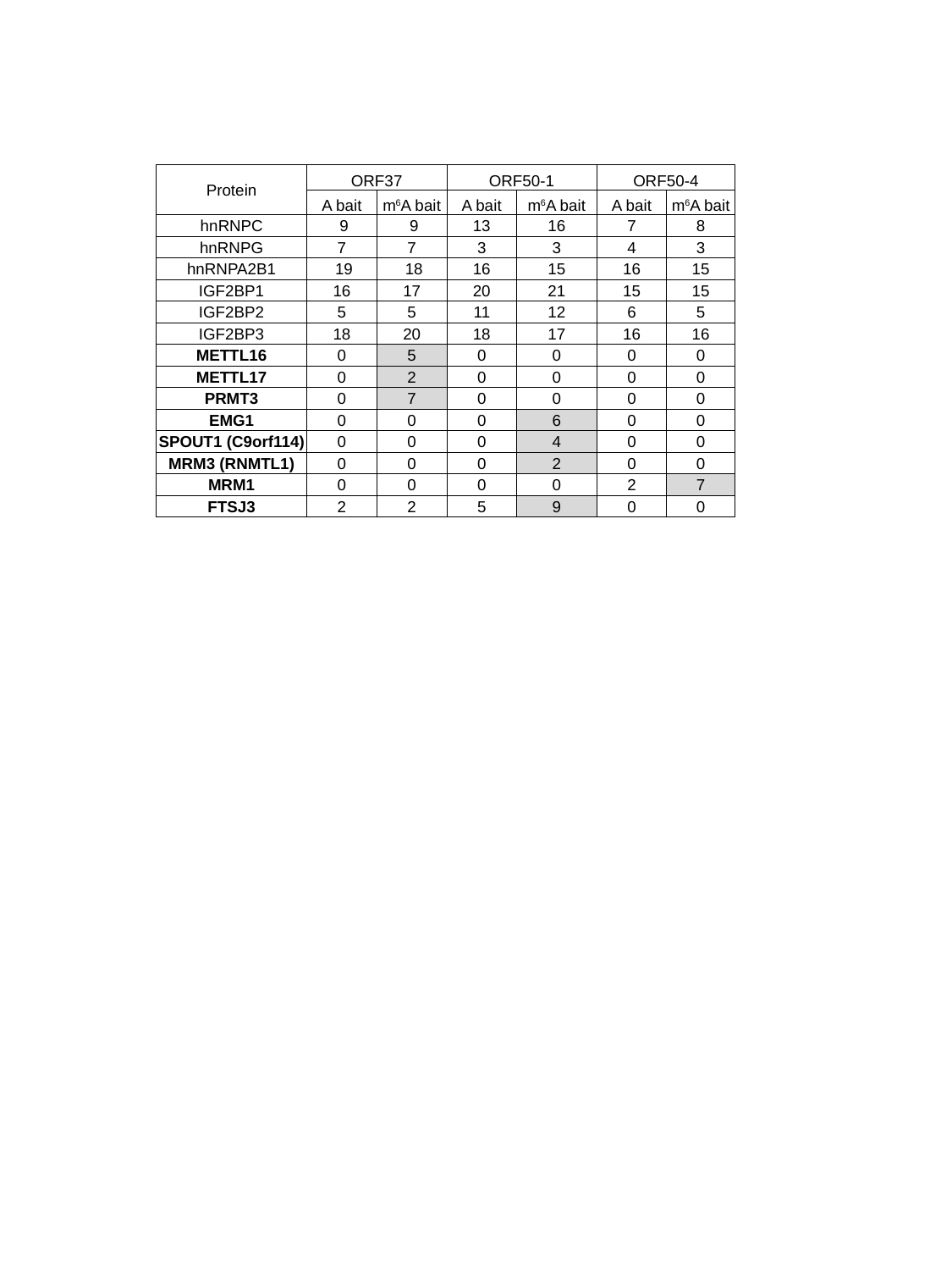

| Protein | ORF37 | | ORF50-1 | | ORF50-4 | |
| --- | --- | --- | --- | --- | --- | --- |
| | A bait | m6A bait | A bait | m6A bait | A bait | m6A bait |
| hnRNPC | 9 | 9 | 13 | 16 | 7 | 8 |
| --- | --- | --- | --- | --- | --- | --- |
| hnRNPG | 7 | 7 | 3 | 3 | 4 | 3 |
| hnRNPA2B1 | 19 | 18 | 16 | 15 | 16 | 15 |
| IGF2BP1 | 16 | 17 | 20 | 21 | 15 | 15 |
| IGF2BP2 | 5 | 5 | 11 | 12 | 6 | 5 |
| IGF2BP3 | 18 | 20 | 18 | 17 | 16 | 16 |
| METTL16 | 0 | 5 | 0 | 0 | 0 | 0 |
| METTL17 | 0 | 2 | 0 | 0 | 0 | 0 |
| PRMT3 | 0 | 7 | 0 | 0 | 0 | 0 |
| EMG1 | 0 | 0 | 0 | 6 | 0 | 0 |
| SPOUT1 (C9orf114) | 0 | 0 | 0 | 4 | 0 | 0 |
| MRM3 (RNMTL1) | 0 | 0 | 0 | 2 | 0 | 0 |
| MRM1 | 0 | 0 | 0 | 0 | 2 | 7 |
| FTSJ3 | 2 | 2 | 5 | 9 | 0 | 0 |
