# Supplementary material for: The Tudor SND1 protein is an m6A RNA reader essential for replication of Kaposi’s sarcoma-associated herpesvirus
Source: eLife. 2019 Oct 24;8:e47261. doi: 10.7554/eLife.47261 (PMC6812964; doi:10.7554/eLife.47261)
Supplement: Supplementary file 3. [file elife-47261-supp3.pptx]

## Slide 1
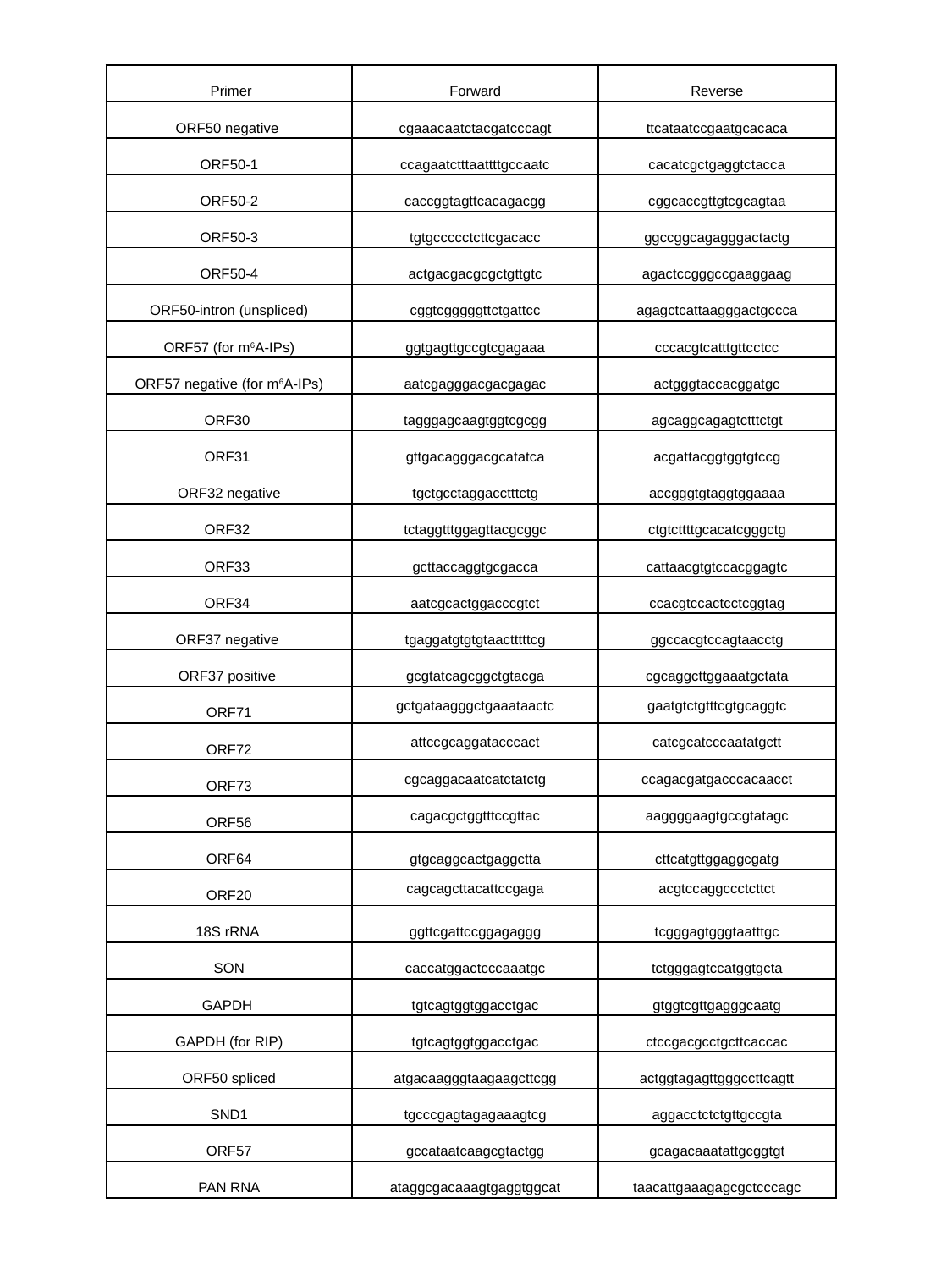

| Primer | Forward | Reverse |
| --- | --- | --- |
| ORF50 negative | cgaaacaatctacgatcccagt | ttcataatccgaatgcacaca |
| ORF50-1 | ccagaatctttaattttgccaatc | cacatcgctgaggtctacca |
| ORF50-2 | caccggtagttcacagacgg | cggcaccgttgtcgcagtaa |
| ORF50-3 | tgtgccccctcttcgacacc | ggccggcagagggactactg |
| ORF50-4 | actgacgacgcgctgttgtc | agactccgggccgaaggaag |
| ORF50-intron (unspliced) | cggtcgggggttctgattcc | agagctcattaagggactgccca |
| ORF57 (for m6A-IPs) | ggtgagttgccgtcgagaaa | cccacgtcatttgttcctcc |
| ORF57 negative (for m6A-IPs) | aatcgagggacgacgagac | actgggtaccacggatgc |
| ORF30 | tagggagcaagtggtcgcgg | agcaggcagagtctttctgt |
| ORF31 | gttgacagggacgcatatca | acgattacggtggtgtccg |
| ORF32 negative | tgctgcctaggacctttctg | accgggtgtaggtggaaaa |
| ORF32 | tctaggtttggagttacgcggc | ctgtcttttgcacatcgggctg |
| ORF33 | gcttaccaggtgcgacca | cattaacgtgtccacggagtc |
| ORF34 | aatcgcactggacccgtct | ccacgtccactcctcggtag |
| ORF37 negative | tgaggatgtgtgtaactttttcg | ggccacgtccagtaacctg |
| ORF37 positive | gcgtatcagcggctgtacga | cgcaggcttggaaatgctata |
| ORF71 | gctgataagggctgaaataactc | gaatgtctgtttcgtgcaggtc |
| ORF72 | attccgcaggatacccact | catcgcatcccaatatgctt |
| ORF73 | cgcaggacaatcatctatctg | ccagacgatgacccacaacct |
| ORF56 | cagacgctggtttccgttac | aaggggaagtgccgtatagc |
| ORF64 | gtgcaggcactgaggctta | cttcatgttggaggcgatg |
| ORF20 | cagcagcttacattccgaga | acgtccaggccctcttct |
| 18S rRNA | ggttcgattccggagaggg | tcgggagtgggtaatttgc |
| SON | caccatggactcccaaatgc | tctgggagtccatggtgcta |
| GAPDH | tgtcagtggtggacctgac | gtggtcgttgagggcaatg |
| GAPDH (for RIP) | tgtcagtggtggacctgac | ctccgacgcctgcttcaccac |
| ORF50 spliced | atgacaagggtaagaagcttcgg | actggtagagttgggccttcagtt |
| SND1 | tgcccgagtagagaaagtcg | aggacctctctgttgccgta |
| ORF57 | gccataatcaagcgtactgg | gcagacaaatattgcggtgt |
| PAN RNA | ataggcgacaaagtgaggtggcat | taacattgaaagagcgctcccagc |

## Slide 2
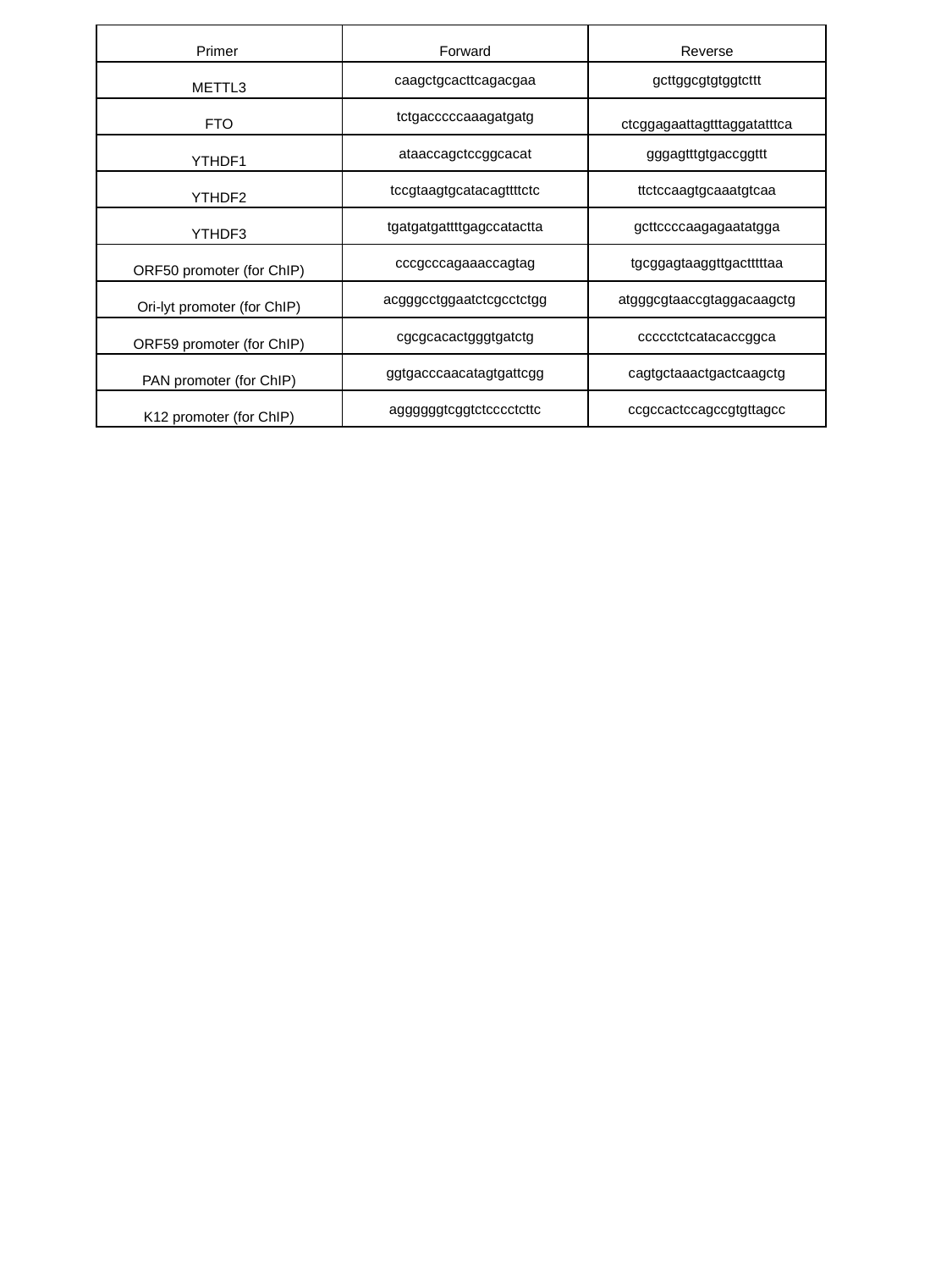

| Primer | Forward | Reverse |
| --- | --- | --- |
| METTL3 | caagctgcacttcagacgaa | gcttggcgtgtggtcttt |
| FTO | tctgacccccaaagatgatg | ctcggagaattagtttaggatatttca |
| YTHDF1 | ataaccagctccggcacat | gggagtttgtgaccggttt |
| YTHDF2 | tccgtaagtgcatacagttttctc | ttctccaagtgcaaatgtcaa |
| YTHDF3 | tgatgatgattttgagccatactta | gcttccccaagagaatatgga |
| ORF50 promoter (for ChIP) | cccgcccagaaaccagtag | tgcggagtaaggttgactttttaa |
| Ori-lyt promoter (for ChIP) | acgggcctggaatctcgcctctgg | atgggcgtaaccgtaggacaagctg |
| ORF59 promoter (for ChIP) | cgcgcacactgggtgatctg | ccccctctcatacaccggca |
| PAN promoter (for ChIP) | ggtgacccaacatagtgattcgg | cagtgctaaactgactcaagctg |
| K12 promoter (for ChIP) | aggggggtcggtctcccctcttc | ccgccactccagccgtgttagcc |
