# Supplementary material for: The Tudor SND1 protein is an m6A RNA reader essential for replication of Kaposi’s sarcoma-associated herpesvirus
Source: eLife. 2019 Oct 24;8:e47261. doi: 10.7554/eLife.47261 (PMC6812964; doi:10.7554/eLife.47261)
Supplement: Transparent reporting form [file elife-47261-transrepform.docx]

***eLife’s* transparent reporting form**

We encourage authors to provide detailed information *within their submission* to facilitate the interpretation and replication of experiments. Authors can upload supporting documentation to indicate the use of appropriate reporting guidelines for health-related research (see [EQUATOR Network](http://www.equator-network.org/%20)), life science research (see the [BioSharing Information Resource](https://biosharing.org/)), or the [ARRIVE guidelines](http://www.plosbiology.org/article/info:doi/10.1371/journal.pbio.1000412) for reporting work involving animal research. Where applicable, authors should refer to any relevant reporting standards documents in this form.

If you have any questions, please consult our Journal Policies and/or contact us: [editorial@elifesciences.org](mailto:editorial@elifesciences.org).

**Sample-size estimation**

- You should state whether an appropriate sample size was computed when the study was being designed
- You should state the statistical method of sample size computation and any required assumptions
- If no explicit power analysis was used, you should describe how you decided what sample (replicate) size (number) to use

Please outline where this information can be found within the submission (e.g., sections or figure legends), or explain why this information doesn’t apply to your submission:

All data was obtained from cellular *in vitro* experiments, thus no sample-size estimation was required. The number of biological replicates is stated on each figure legend when required.

**Replicates**

- You should report how often each experiment was performed
- You should include a definition of biological versus technical replication
- The data obtained should be provided and sufficient information should be provided to indicate the number of independent biological and/or technical replicates
- If you encountered any outliers, you should describe how these were handled
- Criteria for exclusion/inclusion of data should be clearly stated
- High-throughput sequence data should be uploaded before submission, with a private link for reviewers provided (these are available from both GEO and ArrayExpress)

Please outline where this information can be found within the submission (e.g., sections or figure legends), or explain why this information doesn’t apply to your submission:

This information can be found within the figure legends. Biological replicates refer to experiments carried out on different days. Independent m^6^A-IPs and RIPs were performed from independent viral reactivations and independent transfections, each immunoprecipitation replicate was carried out independently on different days.

All deep-sequencing data discussed in this publication have been deposited in NCBI’s GEO Database, GEO accession number GSE119026. All identified peptides/PSMs for each RNA bait by mass spectrometry can be found in the Supplementary Data files.

**Statistical reporting**

- Statistical analysis methods should be described and justified
- Raw data should be presented in figures whenever informative to do so (typically when N per group is less than 10)
- For each experiment, you should identify the statistical tests used, exact values of N, definitions of center, methods of multiple test correction, and dispersion and precision measures (e.g., mean, median, SD, SEM, confidence intervals; and, for the major substantive results, a measure of effect size (e.g., Pearson's r, Cohen's d)
- Report exact p-values wherever possible alongside the summary statistics and 95% confidence intervals. These should be reported for all key questions and not only when the p-value is less than 0.05.

Please outline where this information can be found within the submission (e.g., sections or figure legends), or explain why this information doesn’t apply to your submission:

Statistical methods are described in the Methods section. Error bars and n number are described in the legends for each figure.

Where significant values (p<0.05), p-values are shown as footnotes in figure legends and indicated as * = p < 0.05, ** = p < 0.01, *** = p < 0.001

(For large datasets, or papers with a very large number of statistical tests, you may upload a single table file with tests, Ns, etc., with reference to sections in the manuscript.)

**Group allocation**

- Indicate how samples were allocated into experimental groups (in the case of clinical studies, please specify allocation to treatment method); if randomization was used, please also state if restricted randomization was applied
- Indicate if masking was used during group allocation, data collection and/or data analysis

Please outline where this information can be found within the submission (e.g., sections or figure legends), or explain why this information doesn’t apply to your submission:

Group allocation or masking were not required for these experiments.

**Additional data files (“source data”)**

- We encourage you to upload relevant additional data files, such as numerical data that are represented as a graph in a figure, or as a summary table
- Where provided, these should be in the most useful format, and they can be uploaded as “Source data” files linked to a main figure or table
- Include model definition files including the full list of parameters used
- Include code used for data analysis (e.g., R, MatLab)
- Avoid stating that data files are “available upon request”

Please indicate the figures or tables for which source data files have been provided:

Raw data for all graphs in both the main and supplementary figures are provided in corresponding Source Data Excel documents.

m^6^A peaks called in TREx BCBL1-Rta cells, cellular SND1 targets in TREx BCBL1-Rta cells identified by transcript-wide analysis of RIP-seq and a list of differential SND1-binding events to target RNAs can be accessed in Supplementary Data Excel files. All identified peptides/PSMs for each RNA bait by mass spectrometry can be found in the Supplementary Data Excel files.
